# Purse-string suture with nylon cords and metal clips for the treatment of duodenal fistulae under the endoscope: a case report

**DOI:** 10.3389/fmed.2024.1403218

**Published:** 2024-06-14

**Authors:** Lei Wang, Ruiya Zhang, Bochu Wang, Guoxiong Zhou, Xiaorong Zhou, Run Meng

**Affiliations:** ^1^Department of Immunology, Medical School, Nantong University, Nantong, Jiangsu, China; ^2^Department of Gastroenterology, Affiliated Hospital of Nantong University, Nantong, Jiangsu, China; ^3^Key Laboratory of Biorheological Science and Technology, Ministry of Education, College of Bioengineering, Chongqing University, Chongqing, China

**Keywords:** duodenal fistulae, purse-string suture, endoscope, minimally invasive treatment, metal clips

## Abstract

Purse-string suture with nylon cords and metal clips under the endoscope is a novel therapeutic technique which is minimally invasive and it is particularly indicated for the closure and repair of gastrointestinal fistula or perforations such as duodenal fistulae. Duodenal fistulae are often caused by medical manipulation, disease progression or trauma. Once this occurs, it leads to a series of pathophysiologic changes and a variety of complications. In most cases, these complications will exacerbate the damage to the organism, and the complications are difficult to treat and can lead to infections, nutrient loss, multi-organ dysfunction and many other adverse effects. In this case report, the use of endoscopic nylon cords combined with purse-string suture and metal clips in the treatment of duodenal fistula is presented and discussed. The patient was treated with endoscopic purse-string suture and the duodenal fistula was significantly improved. The results indicate that endoscopic purse-string suture is an effective strategy for the treatment of duodenal fistulae.

## Introduction

The duodenum is an inter-peritoneal organ adjacent to the liver, gallbladder, stomach, jejunum, transverse colon and the right kidney and ureter (1, 2). All of these related surgeries on adjacent organs can result in damage to the duodenum (3). Failure to provide prompt management after duodenal damage will result in severe abdominal infection and systemic inflammatory responses. In the development of modern medical technology, endoscopic minimally invasive treatment is increasingly favored for its advantages of less trauma and faster recovery (4–6). Among them, nylon cords, as a kind of medical polymer material, play an increasingly important role in modern minimally invasive gastrointestinal endoscopic surgery (7, 8). Nylon cords combined with purse-string suture and metal clips is a special strategy that guides the nylon cords around the perforation site to form a structure similar to a “purse-string” under endoscope, and then tightening the suture and fixing it with metal clips to close the perforation, isolating the source of contamination, and promoting the healing of the wound. This approach takes full advantage of the good biocompatibility, flexibility and tensile strength of nylon cords to stably close the perforated area without adding too much trauma. In practice, endoscopic fixation devices such as metal clips are usually used in conjunction with the initial fixation of the perforation edges, followed by a more secure purse-string suture using a nylon cord, thus achieving double-insurance closure of gastrointestinal perforations and reducing the risk of surgical failure and recurrence.

## Case presentation

The patient is a 19-year-old woman, and she was in good health in the past. The patient developed right upper abdominal pain with paroxysmal colic, accompanied by radiating pain in the right shoulder without any obvious causes 3 months ago, and abdominal ultrasound suggested gallbladder stones with cholecystitis. She did not receive treatment at first, then the pain worsened and she was admitted to the hospital because of recurrent right upper abdominal pain for more than 3 months. After admission, magnetic resonance cholangiopancreatography (MRCP) showed multiple gallbladder stones, cholecystitis, acute pancreatitis with exudation and abdominal effusion. It was also accompanied by abnormal biochemical parameters. The proposed admission was followed by cholecystectomy, and the diagnosis of acute pancreatitis was made in conjunction with ancillary tests. The patient’s symptoms were relieved after treatment with antibiotics and inhibition of pancreatic enzyme secretion. Laparoscopic cholecystectomy was performed for further treatment after ruling out contraindications to surgery. During the operation, dense tissue was found in the gallbladder triangle, and the whole layer of the gallbladder plate was edematous. After stripping the gallbladder, bile overflowed from the liver near the gallbladder triangle, and dissection of the right hepatic duct revealed that the wall of the right hepatic duct was broken in the section of the right hepatic duct toward the hepatic hilum, which was considered to be adhesion between the right hepatic duct and the wall of the gallbladder, so repair of the lateral wall of the right hepatic duct was carried out, and a drainage tube was put in place for draining the bile. After surgery, the subhepatic drain drained about 100 mL of light brown fluid per day and the subhepatic negative pressure drain drained 10 mL of light brown fluid, which could be due to bile leakage. After treatment with antibiotics and antispasmodic therapy, the symptoms showed significant improvement.

**Figure 1:**
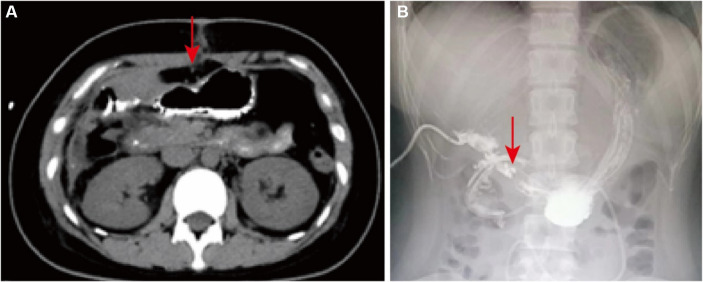
Diagnostic results. **(A)** The abdominal CT image. The red arrow points to pneumoperitoneum. **(B)** The upper gastrointestinal imaging finding. The red arrow points to the fistula.

After 12 days, the patient’s subhepatic drain was draining approximately 50 mL of bile per day, and a repeat MRCP demonstrated a right posterior hepatic duct variant and right posterior hepatic duct injury. Abdominal computed tomography (CT) showed trace pelvic effusion after gallbladder surgery. A consultation with an outside specialist concluded that the bile leak was unlikely to disappear on its own and that an elective partial hepatectomy should be performed. After surgery, the right subdiaphragmatic drain drained about 5 mL of light red fluid per day, and the pelvic drain drained about 10 mL of light red fluid per day. The patient had low-grade fever and was considered to have a high possibility of bile leakage, and was treated with antibiotics, acid suppression, gastric protection, and nutritional support, and the drains were removed sequentially. Repeat abdominal CT demonstrated a massed and flaky pneumatoconcentric shadow, fluid flatness, and striated hyperdense shadow below the liver and in the surgical area after gallbladder surgery. This may be due to fluid and gas retention. Other symptoms were present, such as localized underglossing of adjacent gastric sinusoids and lateral wall of duodenum, fatty liver and a small amount of fluid accumulated in the abdominopelvic cavity (Figure 1A). CT images of the patient showed gas and fluid in the liver area, which may have been caused by poor drainage. The puncture and drainage procedure was performed under the guidance of CT images, and 100 mL of turbid fluid was drained. A drain was placed to continue drainage and approximately 120 mL of fluid was drained after 12 h. For verification of the possibility of a duodenal fistula, ascites was sent to be examined for amylase, and upper gastroenterography was suggestive of a duodenal fistula (Figure 1B).

**Figure 2:**
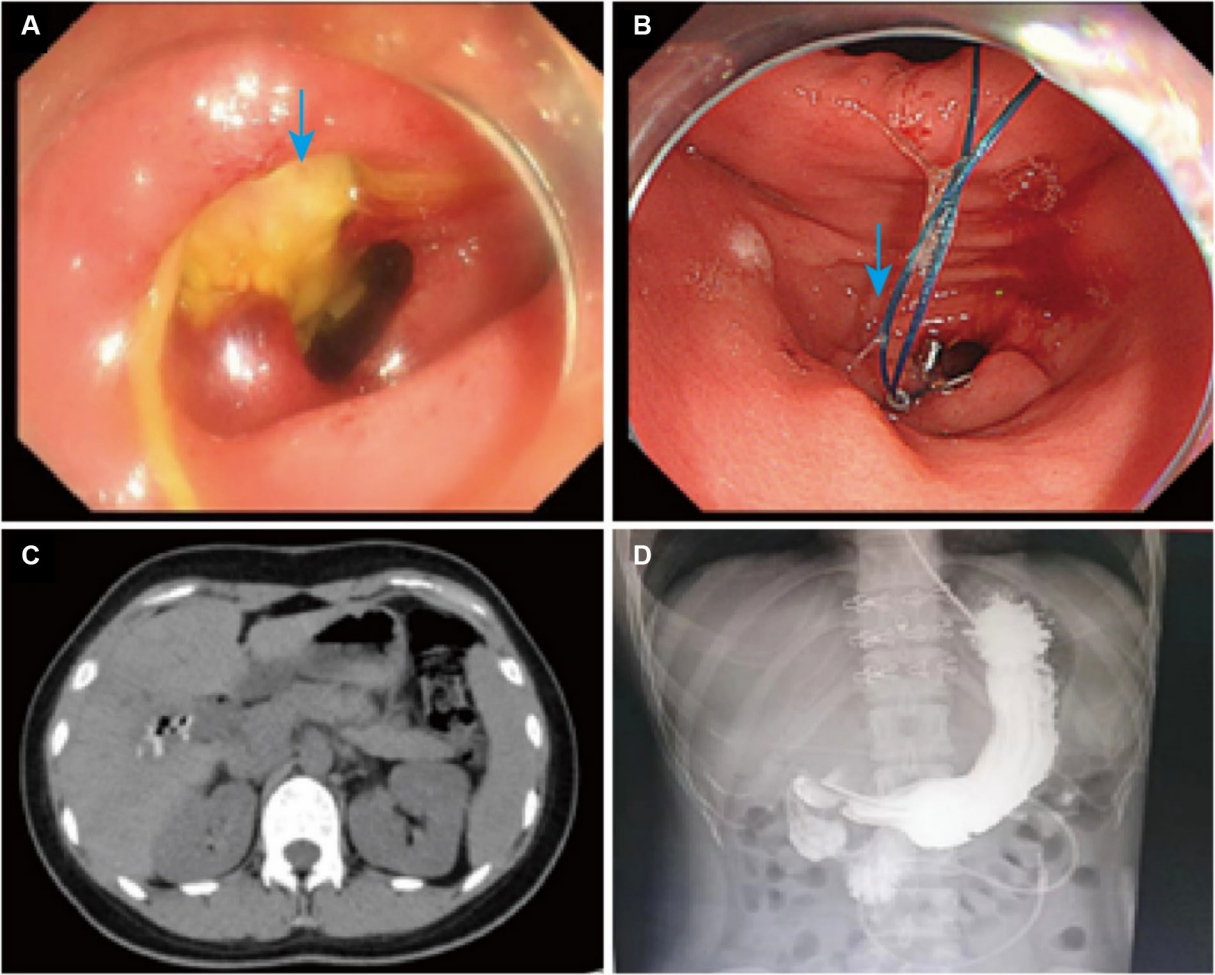
Changes before and after treatment. **(A)** Physical picture before treatment. The blue arrow points to the duodenal fistula. **(B)** Physical picture after treatment. The blue arrow points to the post-surgical trauma. **(C)** The post-treatment CT image of abdomen. **(D)** The upper gastrointestinal imaging result after treatment.

Considering the biliary fistula as the cause of duodenal erosion, gastroscopy was performed to rule out underlying duodenal disease. After a clear diagnosis, a nasoenteric nutritional tube was placed under gastroscopy to the junction of duodenum and jejunum, and at the same time, the duodenal fistula was operated by purse-string suture with nylon cords, and nasal feeding was given after the operation, and the patient was discharged after recovery (Figures 2A,B). Twenty days later, the patient was readmitted to the hospital for the discovery of a duodenal fistula for more than 1 month, and a repeat abdominal CT demonstrated hyperdense shadows at the lower edge of the liver and a slight resorption of gas compared to the previous contrast, and there was also a small amount of abdominopelvic effusion after gallbladder and duodenal surgery (Figure 2C). Upper gastrointestinal imaging showed no significant duodenal fistula (Figure 2D). The overall condition of the patient at follow-up was good.

## Discussion

A duodenal fistula is an abnormal passage formed between the duodenum and other organs or on the surface of the body that causes the contents of the intestinal lumen to flow into the abdominal cavity or outside of the body, usually resulting in severe infections, electrolyte disturbances, malnutrition, and other complications (9). Causes of duodenal fistula include medical, traumatic, tumor, tuberculosis, etc., of which medical causes account for about 80% (10–12). For common medical duodenal fistula, surgical treatment is usually the mainstay, and some patients may choose conservative treatment. Conservative treatment includes drainage, correction of water and electrolyte disorders, infection control, nutritional support and other comprehensive treatment (13). Conservative treatment is generally indicated for those with mild symptoms and small fistulas, and is associated with long hospitalization and complications (14). The traditional surgical approach focuses on trimming the area of inflammatory necrosis at the edge of the duodenum and repairing it with complementary internal and external drainage (15). If the repair is unsatisfactory, resection of the sinus and duodenal bulb with gastrojejunal anastomosis may also be performed. For injuries to the descending and horizontal portions of the duodenum, a Roux-en-Y anastomosis of the jejunoduodenal injury can also be performed. In this case, a series of changes in the patient’s condition resulted in a duodenal fistula, and she was discharged from the hospital with symptomatic improvement after treatment with nylon cords combined with metal clips and loaded sutures (16–18).

For endoscopic fistula or perforation of the digestive tract, there are simple metal clip closure, rake metal clip closure system, nylon cords combined with metal clip loaded suture, new suture equipment OVERSTICH and so on (19–21). Currently, there are 3 main problems with metal clip closure of wounds. First is that metal clips have a limited diameter. Second is that metal clips are not suitable for larger wounds. The last is that the use of metal clips can easily lead to complications. At the 2003 American Digestive Disease Week Meeting, Japanese scholars first described the effective closure of mucosal injury after endoscopic mucosal resection (EMR) by nylon cords combined with metal clips (22). Nylon cords have been used as an auxiliary suturing technology in endoscopic submucosal dissection (ESD), EMR, endoscopic full-thickness resection (EFTR), fistula closure, perforation closure, foreign body removal and other clinical operations are gradually popularized (23–26). Nylon cords combined with purse-string suture and metal clips can be used to suture or bind the tissue around the fistula to create a localized purse-string effect which effectively controls the spread of infection and promotes closure of the fistula.

The specific operation of the nylon cords combined with purse-string suture and metal clips is as follows:

1. According to the patient’s specific situation, endoscope is performed to determine the location, size and surrounding tissue of the fistula. From the endoscopic biopsy channel, the metal clip is inserted, and the open nylon cord is clamped, the nylon cord is pushed to the traumatic surface, the handle of the nylon cord is kept still, and the nylon cord continues to be opened.
2. The is slowly backed up, and the cord is pushed at the same time until the nylon cord is completely exposed to the field of view.
3. Adjusting the angle of the metal clip and bringing the nylon cord to the distal edge of the trauma for clamping and fixation, continuing to use the metal clip to hold the nylon cord at the remaining edges of the trauma.
4. Slowly tightening the handle and straightening the tilted metal clip, lifting the nylon cord and tightening the trauma for closure. And finally, inspecting the trauma and adding additional clips, if necessary, until the trauma is completely closed.

**Figure 3:**
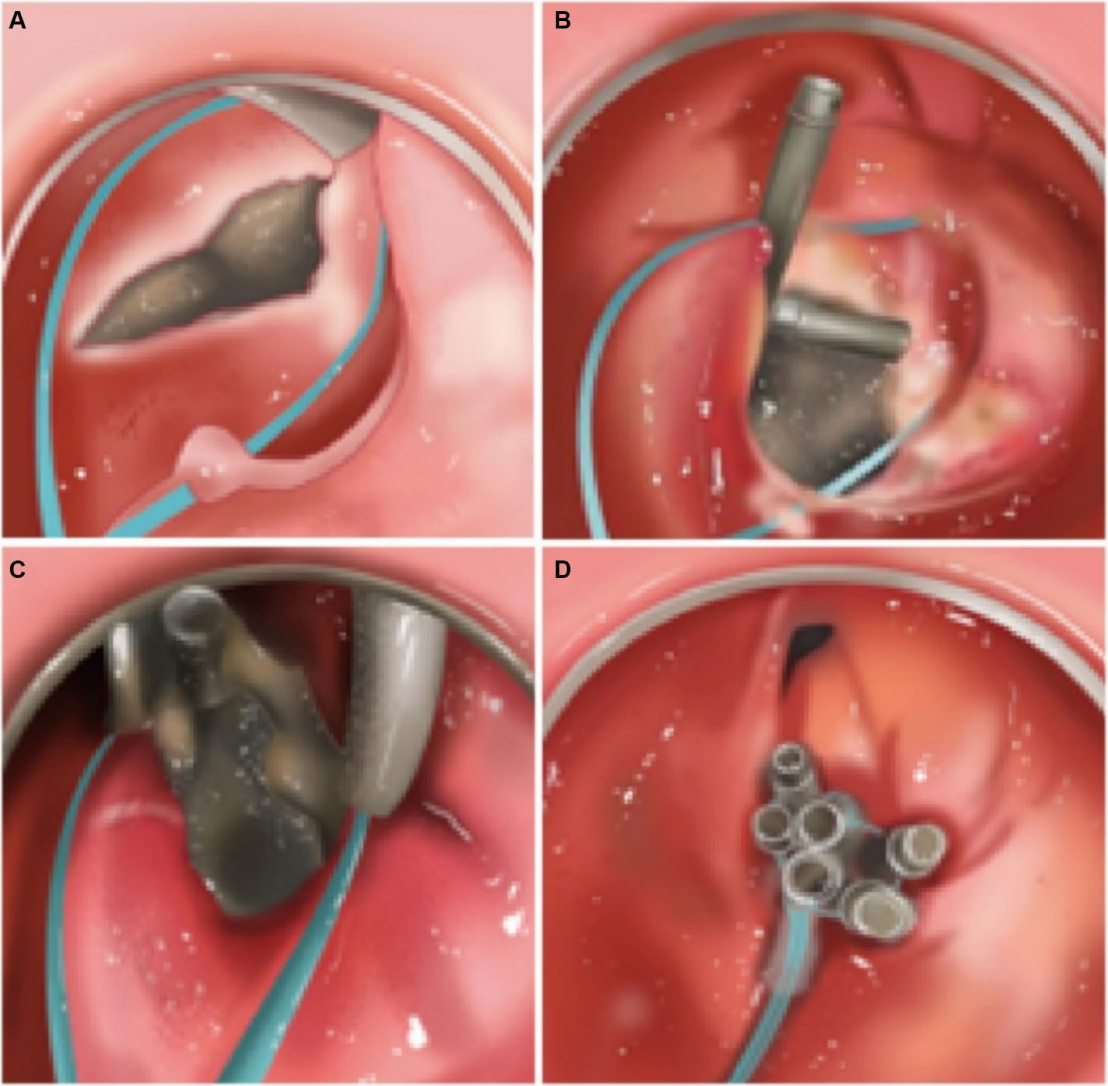
The schematic diagram of nylon cords combined with purse-string suture and metal clips. **(A)** Placement of nylon cords. **(B)** Initial fixation with the metal clip. **(C)** Making the metal clip closed in sequence starting from the edges. **(D)** Tightening and close.

Figure 3 is the schematic diagram of procedure of nylon cords combined with purse-string suture and metal clips.

The advantages of nylon cords combined with purse-string suture and metal clips are mainly reflected in the following aspects:

1. Minimally invasive: Compared with traditional open surgery, this surgical method preserves the original physiological anatomy of the patient, and it greatly reduces the surgical trauma, and is conducive to the rapid recovery of the patient.
2. Safety: The use of nylon cords and metal clips improves the stability of wound closure and reduces the risk of surgical failure and complications.
3. Ease of operation: With the advanced endoscopic technology, the surgeon can operate precisely under direct vision, improving the success rate of the surgery.
4. High efficiency: The surgical method can quickly and effectively close the perforation or fistula, reduce the rate of deterioration, and facilitate subsequent treatment and care.

## Conclusion

In this case report, a novel strategy was used to treat duodenal fistulae. Patients are significantly improved by purse-string suture with nylon cords and metal clips under the endoscope. The clinical research demonstrates that endoscopic nylon cords combined with purse-string suture and metal clips is easy to operate and has a wider range of clinical applications, it also can effectively reduce the occurrence of postoperative complications, and it is worthy of clinical promotion and application.

## Data availability statement

The original contributions presented in the study are included in the article/supplementary material, further inquiries can be directed to the corresponding authors.

## Ethics statement

The studies involving humans were approved by Affiliated Hospital of Nantong University. The studies were conducted in accordance with the local legislation and institutional requirements. Written informed consent for participation in this study was provided by the participants’ legal guardians/next of kin. Written informed consent was obtained from the individual(s) for the publication of any potentially identifiable images or data included in this article. Written informed consent was obtained from the participant/patient(s) for the publication of this case report.

## Author contributions

LW: Writing – original draft, Investigation, Formal analysis, Data curation. RZ: Writing – review & editing, Methodology, Data curation. BW: Methodology, Writing – review & editing. GZ: Formal analysis, Funding acquisition, Project administration, Supervision, Writing – review & editing. XZ: Formal analysis, Funding acquisition, Project administration, Supervision, Writing – review & editing. RM: Conceptualization, Formal analysis, Investigation, Methodology, Writing – original draft, Writing – review & editing.
